# Second‐Harmonic Hyper‐Mie Optical Activity Enables Closed‐Loop Chiral Photochemistry

**DOI:** 10.1002/adma.73593

**Published:** 2026-06-11

**Authors:** Hoyeon Choi, Kody Whisnant, Ben J. Olohan, E. Petronijevic, G. Dan Pantoș, Nicholas A. Kotov, Ventsislav K. Valev

**Affiliations:** ^1^ Centre For Photonics Department of Physics University of Bath Bath UK; ^2^ Department of Chemical Engineering University of Michigan Ann Arbor Michigan USA; ^3^ Biointerfaces Institute University of Michigan Ann Arbor Michigan USA; ^4^ SBAI Department La Sapienza University of Rome Rome Italy; ^5^ Department of Chemistry University of Bath Bath UK; ^6^ Department of Electronic & Electrical Engineering University of Bath Bath UK

**Keywords:** chirality, nonlinear optics, optical activity, photochemistry

## Abstract

Photochemistry promises sustainable chemical processing but typically depends on ultraviolet light with limited selectivity and penetration. We report nonlinear chiral photochemistry, where femtosecond infrared pulses are frequency‐doubled to both drive and track a transformation of chiral CdTe/CdO nanohelices into CdO nanospheroids. Circularly polarized light induces a controlled oxidation sequence monitored in real time through second‐harmonic scattering intensity and chiroptical contrast. As the CdO shell fractures and exposes non‐centrosymmetric CdTe, second‐harmonic intensity rises twenty‐fold, polarization reverses, and characteristic CdTe photoluminescence emerges. These findings are enabled by the experimental observation of the second‐harmonic hyper‐Mie optical activity effect, which completes a suite of nonlinear chiroptical scattering phenomena predicted over 45 years ago. Our results offer a spatially confined, selective, and temporally‐resolved method for material transformation.

## Graphical abstract

Nonlinear chiral photochemistry is demonstrated in CdTe/CdO nanohelices, where circularly polarized infrared light both drives and tracks photo‐oxidation via second‐harmonic scattering. This closed‐loop process reveals real‐time structural evolution, including shell fracturing and core exposure, accompanied by a transition from hyper‐Mie to hyper‐Rayleigh scattering, a reversal of chiroptical response, and the observation of second‐harmonic hyper‐Mie optical activity.

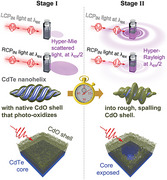

## 1 Main Text

Photochemistry is widely employed in synthesis of organic materials and intermediates, with established applications including photolithography [1, 2], UV curing [3], organic synthesis [4], and photochromic materials [5, 6]. However, most of these processes rely on ultraviolet light, which has limited penetration depth and is often too energetic, causing unwanted side reactions, and photodegradation of targeted products [7]. UV light is also harmful to the eyes and skin. Most of these disadvantages can be mitigated with nonlinear photochemistry using low energy infrared (IR) photons.

Nonlinear photochemistry uses the near‐simultaneous absorption of two or more photons to initiate chemical reactions, typically via IR excitation [8]. These processes involve higher‐order light–matter interactions, described by hyperpolarizability tensors. Accordingly, nonlinear light scattering processes are referred to as hyper‐Rayleigh or hyper‐Mie, for scatterers much smaller and much larger than the wavelength, respectively. Unlike linear photochemistry, which relies on single‐photon absorption, nonlinear photochemistry allows for spatially confined reactivity (at the laser focus), ultrafast temporal resolution, and reduced off‐target activation [9]. At the same time, the requirement for high peak intensities and tight focusing limits the interaction volume and makes bulk‐scale processing challenging. Nonlinear photochemistry has been widely used for photopolymerization [10] and crosslinking [11], photo‐release of biologically active molecules [12], bond cleavage [13], and to drive excited‐state dynamics [14]. However, it has rarely been employed for inorganic compounds. Its application to chiral systems remains virtually unexplored. Here, we apply nonlinear photochemistry to drive the oxidation of CdTe/CdO core/shell nanohelices into CdO, and to track the process in real time through chiroptical second‐harmonic scattering.

Guided by chiral molecules, CdTe nanoparticles (NPs) can assemble in well‐defined chiral shapes–nanohelices [15, 16, 17]. These complex particles interact strongly with circularly polarized light to produce dramatic chiroptical effects in the linear [18], and nonlinear optical regime [19]. Moreover, it has been shown that circularly polarized light can control the assembly of CdTe NPs into chiral structures [20]. CdTe nanohelices now enable, for the first time, the demonstration of nonlinear chiroptical photochemistry.

Chiroptical effects in the harmonics of scattered light were first predicted between 1977 and 1979 [21, 22], however they remained unobserved for over four decades. In nonlinear optics, the even and odd harmonics are completely distinct effects, as they obey different selection rules. Hyper‐Rayleigh scattering optical activity has now been demonstrated at the second‐ [23] and third‐harmonic frequencies [24]. Hyper‐Mie optical activity was demonstrated at the third‐harmonic [19], but there has not been a report of the second‐harmonic effect.

Here, we present a closed‐loop nonlinear chiral photochemical process that transforms CdTe/CdO core/shell nanohelices into CdO. To initiate it, we demonstrate the second‐harmonic hyper‐Mie optical activity effect – illuminating the nanohelices at 730 nm with circularly polarized light results in different second‐harmonic scattering intensity (at 365 nm), depending on the handedness of the illumination and of the nanohelices. Following prolonged illumination, the nonlinear optical interaction then drives a chemical transformation to CdO, which can be followed optically, in real time. Initially, the properties of the thickening centrosymmetric CdO shell dominate the optical response; the refractive index $n_{CdO}^{710-750nm}\approx 1.8$ [25]. Due to laser illumination, shell fractures and scanning electron microscopy reveals a dramatic increase in surface roughness. Once the CdTe core is exposed, the effective refractive index increases ($n_{CdTe}^{710-750nm}\approx 3.0$) [26], which leads to an increase in linear optical scattering. As CdTe is non‐centrosymmetric (zincblende structure), the second‐harmonic intensity increases dramatically (>20 times). Moreover, the second‐harmonic polarization also transitions, becoming much more circular. We also record the characteristic Z‐band photoluminescence of CdTe at 1.36 eV (∼910 nm). In the final CdO state, the particles are fully centrosymmetric and the nonlinear chiroptical signal drops; the refractive index decreases and the linear scattering collapses; in the absence of CdTe, the Z‐band photoluminescence ceases; the particles’ shape becomes spheroidic. The transition to a cracked CdO shell with exposed CdTe core also leads to a significant change in the effective crystallographic order of the scatterers, which can be observed in the polarization state of second‐harmonic light. In demonstrating this behavior, we introduce here a dual CPL (circularly polarized light) chiroptical harmonic scattering configuration, that is conceptually analogous to Nafie introducing dual CPL to Raman optical activity [27] that Barron had initially discovered and demonstrated for single CPL [28].

**FIGURE 1:**
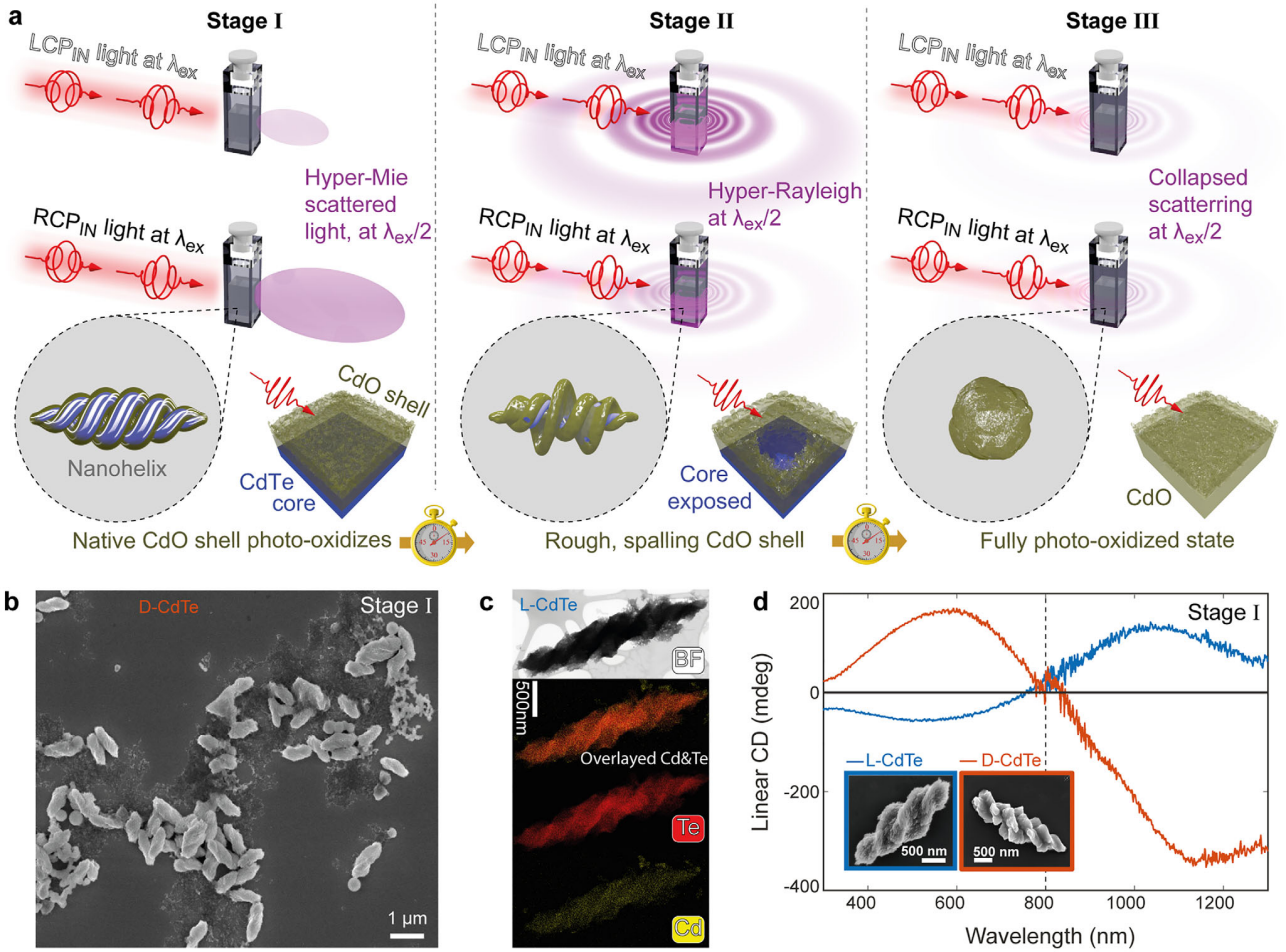
Hyper‐Mie optical activity and nonlinear photochemistry proceed from CdTe nanohelices across three stages. (a), CdTe/CdO nanohelices (∼3 µm) in water scatter second‐harmonic light under femtosecond excitation. The scattering depends on circular polarization and reverses with helix handedness, revealing hyper‐Mie optical activity. Prolonged illumination leads to three stages: in stage I, the CdTe core remains fully enclosed by a continuous CdO shell; in stage II, shell cracking exposes the CdTe core and the scattering transitions into the hyper‐Rayleigh regime with reversed contrast; and in stage III, the particles are fully oxidized into spheroidal CdO NPs. λ_ex_: excitation wavelength; LCP_IN_ & RCP_IN_: incident left‐ and right‐handed circularly polarized light, respectively. (b), Scanning electron microscopy image of D‐CdTe nanohelices shows the typical distribution of sizes. (c), BF: bright field optical microscopy image. Energy‐dispersive x‐ray spectroscopy (EDS) images of the helices reveal the Cd and Te content. L‐CdTe: left‐handed CdTe nanohelix. (d), Circular dichroism (CD) spectra, performed in the linear optical regime, reveal a broad bisignate chiroptical response. In inset, scanning electron microscopy (SEM) images of the CdTe nanohelices; D‐CdTe: right‐handed CdTe nanohelices. Some top‐bottom asymmetry of the spectra are typical for microscale nanostructures due to kinetic effects and strong higher order interaction between NPs during the synthesis.

Figure 1a illustrates the main results, summarized in three stages. In stage I, CdTe/CdO core/shell nanohelices (∼3 µm in length) are dispersed in water and placed in an optical cuvette. When illuminated with femtosecond laser pulses, at wavelength λ_ex_, the nanohelices scatter light at the second‐harmonic (λ_ex_/2) wavelength, preferentially in the forward direction. Moreover, depending on incident right‐ or left‐handed circularly polarized light (RCP_IN_ & LCP_IN_, respectively), the scattered light is more or less intense. The difference in scattered‐harmonic intensity reverses, when the handedness of the CdTe nanohelices reverses, which demonstrates the hyper‐Mie optical activity effect at the second‐harmonic. Following prolonged illumination, the CdO shell thickens. At stage II, it cracks, with spalling and flaking off that expose parts of the CdTe core. The exposed regions have large nonlinearity and their size is smaller than the wavelength, hence the scattering enters the hyper‐Rayleigh regime. In this regime, the chiroptical contrast is reversed. At stage III, the oxidation is almost complete and yields spheroidal CdO NPs.

Figure 1b shows a scanning electron microscopy (SEM) image of D‐CdTe nanohelices with a representative distribution of sizes. Figure 1c presents the element composition in L‐CdTe. Both Cd (in yellow) and Te (in red) are shown in the energy‐dispersive x‐ray spectroscopy (EDS) images. The linear chiroptical behavior for L‐ and D‐CdTe nanohelices is displayed in Figure 1d. The graphs show circular dichroism (CD) as a function of wavelength. The spectrum is bisignate with a crossing around 800 nm. The morphology of both helices is visible in the inset. L‐ and D‐cysteine bias the assembly toward opposite handedness, however the mesoscale self‐assembly of CdTe NPs is not strictly deterministic. Previous studies of these systems have shown that helix formation proceeds through kinetically controlled pathways with significant structural variability, including differences in pitch, thickness, and morphology within nominally homochiral samples [20]. The difference in the CD spectra in Figure 1d is therefore not unexpected.

**FIGURE 2:**
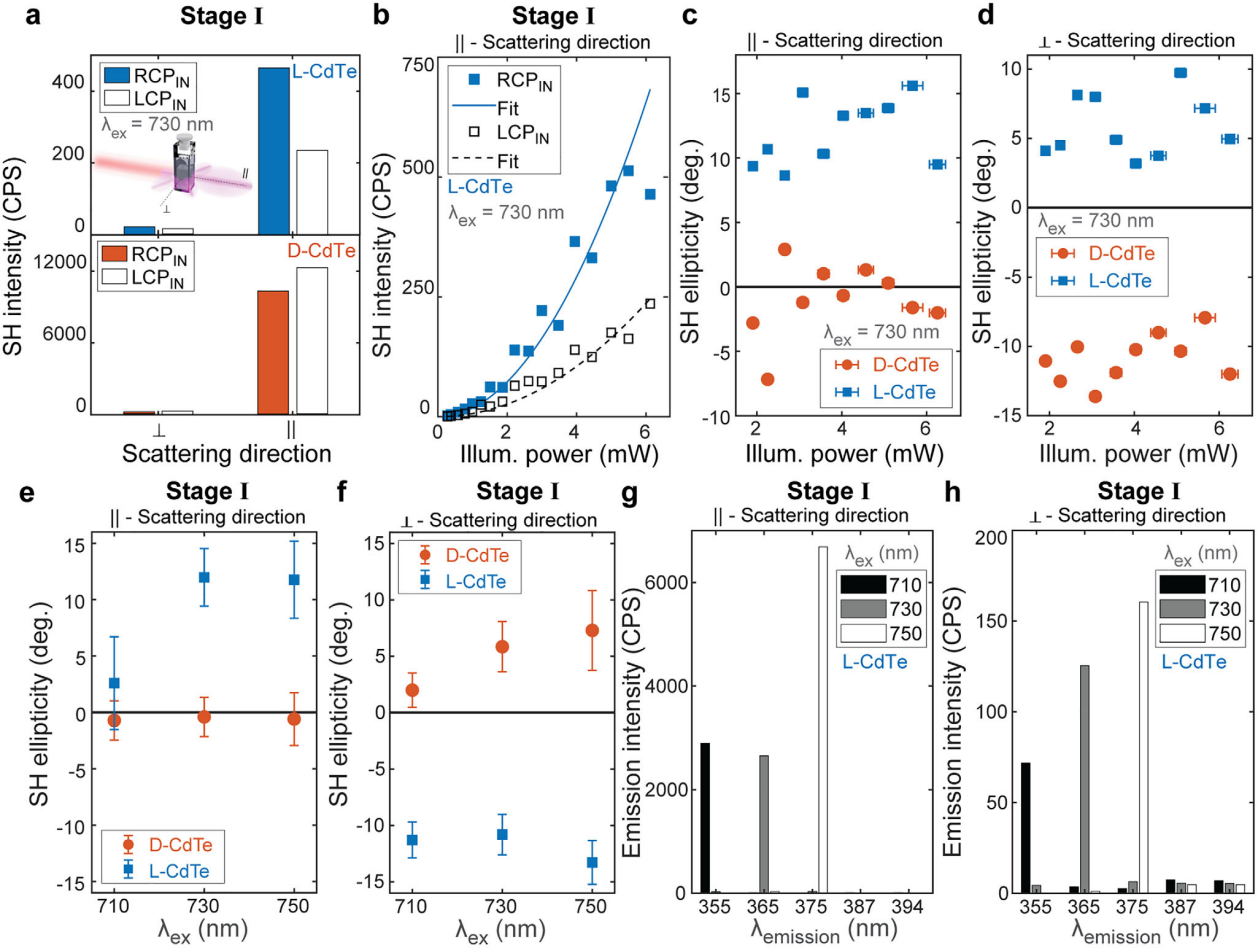
Second‐harmonic hyper‐Mie scattering optical activity, at Stage I. (a), Second‐harmonic (SH) scattered light from L‐CdTe, emitted in the forward (||) and right‐angled (⊥) directions, for incident right‐ and left‐hand circularly polarized light (RCP_IN_ & LCP_IN_, respectively), upon illumination at 730 nm with ∼5 mW incident power. SH intensity dominates in the || direction compared to ⊥, consistently with hyper‐Mie scattering. (b), SH intensity vs. illumination power for RCP_IN_ and LCP_IN_ follows a quadratic law (fits to ***y *** = *** Ax*** ^2^) and exhibits large chiroptical contrast. The nonlinear ellipticity plotted vs. illumination power clearly distinguishes L‐ from D‐CdTe, both in the || and ⊥ directions, shown in (c) and (d), respectively. The average SH ellipticities at three different wavelengths clearly distinguish L‐ from D‐CdTe, both in the || and ⊥ directions, as shown in (e) and (f), respectively. (g) and (h), Multiphoton emission spectra in the || and ⊥ directions, respectively, for excitation wavelengths (***λ***_***ex***_) of 710 nm (black), 730 nm (grey) and 750 nm (white) reveal a very low multiphoton background, compared to the SH signals.

Figure 2 demonstrate the second‐harmonic hyper‐Mie optical activity effect that occurs at stage I. Figure 2a shows the intensity of second‐harmonic scattering in the forward direction (||) and at a right angle (⊥), for L‐CdTe (top panel) and D‐CdTe (bottom panel); the experimental setup is shown in Figure S1. In both samples, much more intensive scattering is observed in the forward direction, which is consistent with Mie‐scattering patterns. Because the size of the structure is comparable to the wavelength, the nonlinear emission from different parts of the helix interferes constructively in the forward direction, leading to strongly directional scattering.

For L‐CdTe, the forward scattering is ∼20 times more in the forward direction. In the case of D‐CdTe, the forward scattering is even more pronounced, likely due to the difference in morphology between L‐ and D‐CdTe. For both samples, the data indicate a clear difference in second‐harmonic intensity depending on the helicity of light and on the chirality of the nanohelices. To illustrate this chiroptical effect, we compare second‐harmonic intensities at different illumination powers.

Figure 2b shows typical power‐dependence curves, here obtained at 730 nm, from L‐CdTe, in the forward direction (the right‐angled scattering and the corresponding curves for D‐CdTe curves are shown in Figure S2). As expected, the second‐harmonic intensity scales quadratically with the incident power (fits to *y* = *Ax* ^2^ yield R^2^ values of >0.93 and of 1, for RCP_IN_ and LCP_IN_, respectively). Importantly, there is a very clear second‐harmonic intensity difference depending on the handedness of illumination. This difference can be quantified using the equation:

(1) $$\mathrm{SH}\;\mathrm{ellipticity}\;=\frac{180}{\pi }\;\arctan\left(\frac{\sqrt{I_{RCP_{IN}}}-\sqrt{I_{LCP_{IN}}}}{\sqrt{I_{RCP_{IN}}}+\sqrt{I_{LCP_{IN}}}}\right)$$

where $I_{RCP_{IN}}$ and $I_{LCP_{IN}}$ are the second‐harmonic intensities obtained for RCP_IN_ and LCP_IN_ illumination, respectively. The SH ellipticity for both samples, in both forward and right‐angled scattering, is shown for each power in Figure 2c,d, respectively. The data are consistent with a well‐pronounced hyper‐Mie optical activity. This effect is robust vs. wavelength change and can be observed at 710 and 750 nm as well (Figure 2e,f, also Figures S3 and S4 for the detailed power‐dependences) as indicated by the mini‐spectra for forward and right‐angled second‐harmonic scattering, respectively. In each case, the two chiral forms of the nanohelices are clearly distinguishable, with a notable reversal of the chiroptical contrast between forward and right‐angled scattering, attributed to the difference in nonlinear hyperpolarizability tensor components involved.

Figure 2g,h shows the multiphoton emission spectra for forward and right‐angled scattering, respectively. In each case, the samples were illuminated with three different wavelengths: 710 nm (black), 730 nm (grey), and 750 nm (white). The spectra correspond to different wavelength filters positioned in front of the detector. In each case, the harmonic scattering signal is far above the multiphoton background, which rules out alternative nonlinear optical effects, such as multiphoton luminescence or supercontinuum emission.

The observation of hyper‐Mie optical activity at the second‐harmonic completes the experimental demonstration of chiroptical harmonic scattering effects in the two main scattering regimes (Rayleigh and Mie), at both even and odd harmonics. We note that between the Rayleigh and Mie scattering, there is less well‐known intermediate scattering regime – often referred to as Tyndall scattering – where nonlinear chiroptical scattering was observed at the second‐harmonic but not yet at the third [29]. Besides the fundamental scientific significance of this hyper‐Mie work, it is also of direct practical importance as it allows to perform closed‐loop nonlinear chiral photochemical processing of high‐value materials.

**FIGURE 3:**
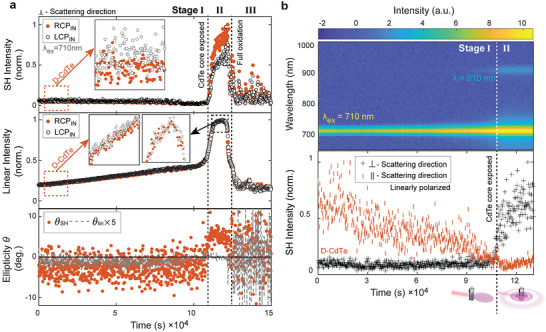
Illumination causes thickening, then cracking of the CdO shell, exposing the CdTe core with high optical nonlinearity, followed by full oxidation. (a), SH (top panel) and linear (middle panel) scattering intensity, as well as SH (***θ***_***SH***_) and linear (***θ***_***lin***_) ellipticities (bottom panel), as a function of illumination time. Illumination was at 710 nm, ∼5 mW average power, with incident right‐ and left‐handed circularly polarized (RCP_IN_ & LCP_IN_, respectively) light. At $∼1.1\times 10^{5}$ s, the scattering increases dramatically, and the SH chiroptical contrast reverses. At $∼1.25\times 10^{5}$ s, the scattering drops and the chiroptical SH contrast disappears. The color scale is logarithmic. (b), In the upper panel, emission spectra from D‐CdTe nanohelices, recorded vs. time. The excitation wavelength (***λ***_***ex***_) of 710 nm is clearly visible. When the CdTe core is exposed, a strong emission at ∼910 nm is recorded, consistent with Z‐band CdTe photoluminescence. In the lower panel, the SH intensity (normalized to the maximum value within each dataset) recorded simultaneously as a function of time. Illumination with linearly polarized light oriented along the vertical (S) direction. While initially SH intensity dominates in the || direction, after the transition, SH intensity dominates in the ⊥ directions, in agreement with a transition from hyper‐Mie to hyper‐Rayleigh scattering, due to a size reduction of the effective scatterers.

Figure 3a presents the scattered light, at right‐angle, from D‐CdTe nanohelices, as a function of illumination time, in both the second‐harmonic (top panel) and the linear (middle panel) optical regimes. D‐CdTe exhibits a lower initial ellipticity than L‐CdTe, nevertheless, the key features of the transformation, including the sign inversion of the chiroptical response, remain clearly observable, demonstrating the robustness of the effect. The illumination wavelength was 710 nm and the average power was ∼5 mW. The bottom panel focusses on the chiroptical behavior, showing the ellipticity calculated for the second‐harmonic (θ_*SH*_) and linear (θ_*lin*_) cases. All panels demonstrate that the D‐CdTe undergoes significant changes over time, consistent with the three stages in Figure 1a. At stage I, in the right‐angled direction, there is very little second‐harmonic scattering light, consistently with hyper‐Mie scattering. A chiroptical effect is still visible, as shown in the inset of the top panel and in the second‐harmonic ellipticity, which is negative on average.

In water, the CdTe nanohelices acquire a thin native CdO shell prior to illumination. Control experiments in which samples were kept in water without laser exposure for comparable durations showed no perceptible structural or optical changes, and no stage‐like transitions. The CdO shell is centrosymmetric with a rock salt crystal structure and a lattice constant of 4.70 Å. Therefore electric‐dipole second‐harmonic is mostly due to the surface and interfaces and it is emitted in the forward direction. As the shell thickens, considerable strain develops at the interface with the CdTe core, whose lattice constant is 6.48 Å. The considerable (25%) mismatch of inorganic lattices causes strain that not only fractures but also produces spalling and flaking off in the shell, leading to stage II.

Stage II starts at ∼1.1 × 10^5^ s, when a sharp increase in the second‐harmonic intensity is observed because of an increase in the surface roughness of the scatterers and because part of the CdTe core becomes exposed to light. CdTe has a zincblende non‐centrosymmetric crystal structure which produces strong second‐harmonic emission. Moreover, CdTe has multiple Mie resonances in the spectral region of the second‐harmonic emission, and these radiate in the right‐angled direction, exactly as we observe. Due to the change in second‐harmonic emission sources, the SH ellipticity reverses sign.

This sign inversion reflects a change in the dominant nonlinear scattering mechanism: from hyper‐Mie scattering (where multiple multipolar contributions interfere) to hyper‐Rayleigh scattering (from smaller, effectively independent emitters). Each scattering regime has its own set of nonlinear tensor components and resulting SH ellipticity. During the following ∼1.25 × 10^5^ s, the second‐harmonic scattered intensity keeps increasing, as the material oxidizes further.

At stage III, the entire scatterer transforms into CdO. As our data show, the second‐harmonic intensity and ellipticity both collapse (and the noise increases dramatically) due to the formation of spherical and centrosymmetric CdO.

A similar behavior can be observed in the linear optical scattering. At stage I, the scattering intensity increases progressively. At stage II, once the CdTe is exposed, the effective refractive index of the scatterer increases, since $n_{CdTe}^{710-750nm}\approx 3.0>n_{CdO}^{710-750nm}\approx 1.8$. The increased surface roughness (shell flaking off) also increases scattering. Eventually, at stage III, the scatter is fully oxidized and reshapes into a smooth sphere (see data below), which causes the collapse of linear optical scattering. To further verify these physical mechanics, we turn to photoluminescence.

Figure 3b shows the photoluminescence spectra of the D‐CdTe nanohelices upon prolonged illumination at 710 nm, while simultaneously collecting second‐harmonic scattering light. At the onset of stage II, strong emission at ∼910 nm is recorded. The emission wavelength and intensity are stable over time. Such emission is consistent with the well‐known 1.36 eV Z‐band photoluminescence in CdTe [30, 31, 32, 33, 34], first reported by Fujii et al. [35] (additional observations of Z‐band photoluminescence onset in Figures S5 and S6). Once the scatterers are almost fully oxidized, CdTe practically disappears and the Z‐band luminescence ceases, see Figure S7.

In the lower panel of Figure 3b, the SH scattered intensity, for S‐polarized illumination, is shown as a function of time, in both the forward and right‐angle directions, across stages I and II. Initially, at stage I, there is much more light scattered forward than at right angle, which is consistent with Mie scattering. At this stage, the nanohelices consist of continuous CdTe covered with a thin shell of CdO. Electric dipoles (E1), electric quadrupoles (E2) and magnetic dipole (M1) all radiate forward within the Mie optical envelope. Moreover, under circularly polarized light, E1‐E1 coupling tensors interfere constructively with E1‐E2 and/or E1‐M2 tensors, which gives rise to the hyper‐Mie optical activity effect. As oxidation proceeds, the CdO shell thickens. Because the thickening CdO is centrosymmetric, the intensity of second‐harmonic light diminishes progressively. At stage II, shell cracks expose ∼5 nm CdTe crystallites, randomly oriented. The subwavelength cracks shift scattering into the hyper‐Rayleigh regime, radiating naturally at right angles, as shown. A quantitative analysis of the transition from forward to right‐angled scattering over the illumination time can be found in Figure S8.

**FIGURE 4:**
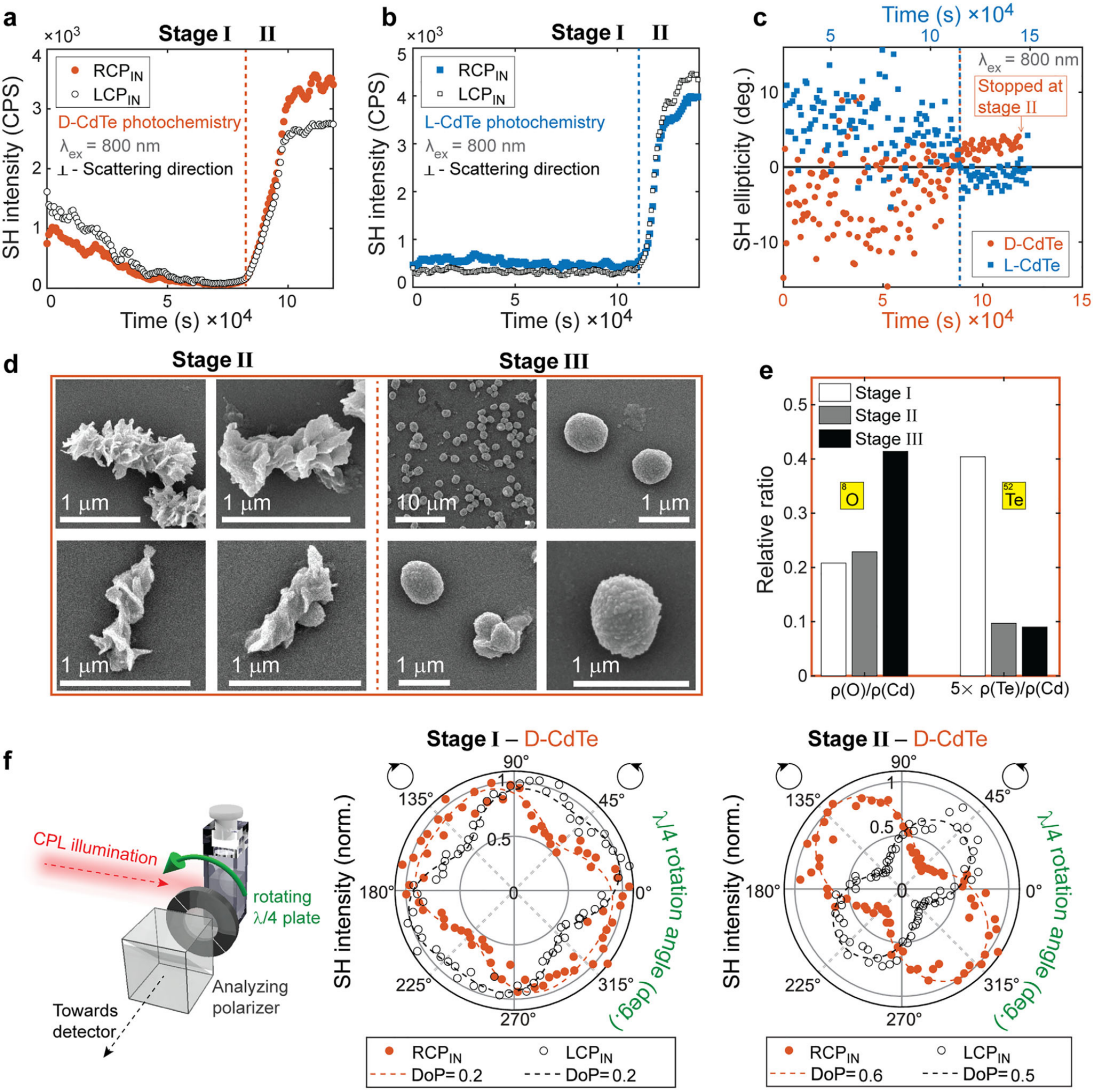
Nonlinear chiroptical photochemistry: geometrical and chemical transformations. (a), Second‐harmonic (SH) intensity scattered from D‐CdTe nanohelices, as a function of time, for incident right‐ and left‐hand circularly polarized light (RCP_IN_ & LCP_IN_, respectively). Illumination at 800 nm, with ∼5 mW and light is detected in the right‐angled (⊥) direction. (b), Similarly organized data for L‐CdTe. A clear chiroptical contrast is visible for both chiral forms of CdTse and, for both, that contrast reverses after the photochemical transformation occurs. (c), The SH ellipticity vs. time demonstrates the chiroptical contrast. To evaluate the material in the transformed state, illumination of the D‐CdTe was stopped at the point indicated with an arrow. (d), Scanning electron microscopy of the NPs collected at stage II reveals pronounced surface roughness, compared to the original nanohelices (see Figure 1). At stage III, the NPs adopt a spherical shape. Scalebars are indicated with white rectangles. (e), Comparison of the Te and O content of the NPs, estimated as a ratio to the Cd. At stage I, prior to illumination (in white), some native oxidation from water/air forms at the surface of the CdTe. At stage II, after the CdO shells thickens and cracks, (in grey), the amount of Te is reduced and the O is increased. At stage III, the ratio of Te to O is almost opposite to that observed stage I, indicating almost complete oxidation of the NPs. (f), The polarization state of SH scattered light at stage I and stage II is very different – the degree of circular polarization (DoP) is larger at stage II – indicating a change in the crystal symmetry of the scatterers.

To demonstrate the sensitivity of the nonlinear chiroptical effect, we next illuminated the samples at 800 nm, where the linear optical spectra are crossing and no CD can be observed, see Figure 1c. Figure 4a,b shows the second‐harmonic scattered intensity as a function of time, in the right‐angle geometry, for RCP and LCP illumination, at an average power of ∼5 mW. At stage I, a clear chiroptical contrast can be observed that reverses as expected, depending on the chirality of CdTe. At stage II, this contrast changes sign for both chiral forms of CdTe. The sign change is also well evidenced by the calculated SH ellipticity, shown in Figure 4c. For L‐CdTe, SH ellipticity switches from positive to negative, and for D‐CdTe it switches from negative to positive. For L‐CdTe, all three stages can be seen in Figure S9. To capture stage II state, D‐CdTe samples were removed for electron microscopy shortly after onset.

Figure 4d shows SEM images of the NPs in stages II and III. At stage II, it is immediately apparent that the surface roughness of the structures is much larger than that of the initial CdTe/CdO core/shall nanohelices. Due to the illumination, CdTe can undergo a two‐photon oxidation process. CdTe absorbs UV photons that generate electron‐hole pairs, i.e. $CdTe+h\nu_{UV}\to e^{-}+h^{+}$, where *h*ν_*UV*_ represents a UV photon, and where *e* ^−^ and *h* ^+^ indicate an excited electron and a hole, respectively. In the presence of water, the Te atoms can be oxidized therefore separating from the parent lattice, e.g. $Te^{2-}+3H_{2}O+6h^{+}\to TeO_{3}^{2-}+6H^{+}$. The Te is oxidized into Te(IV) species (i.e. tellurium in the +4 oxidation state, e.g. TeO_2_ or TeO_3_^2^^−^) and removed into the surrounding solution [36]. At the same time, *O* ^2 −^ replaces *Te* ^2 −^, resulting in CdO. As a result, the CdO shell grows until the building up strain causes cracks and flaking off. Accordingly, multiple 10–30 nm thick flakes can be seen in the SEM images. At stage III, the oxidation causes restructuring and comparatively smooth spheroidal NPs of CdO form, as shown in the SEM images.

The oxidation process is confirmed by energy dispersive x‐ray analysis (EDX), see Figure 4e. Because the amount of Cd remains the same during the oxidation of CdTe, we examine the content of Te and O as a ratio to the measured Cd. At stage I, for CdTe nanohelices dispersed in water, native oxide forms spontaneously at the surface but its thickness is limited. The ratios of native O to Cd and Te to Cd are shown with white bars. At stage II, the amount of Te is significantly reduced, compared to the amount of O, as shown with grey bars. At stage III, after almost complete oxidation, the amount of O far exceeds the Te, as shown in the black bars.

In this and previous works so far, the SH chiroptical scattering measurements have been limited to intensity. However, chiroptical information can also be extracted from the polarization state of the scattered light. The experimental logic parallels that of Raman Optical Activity (ROA), where Barron first demonstrated chiroptical contrast using a single circular polarization, and Nafie later introduced a dual‐CPL scheme that greatly improved experimental robustness [27]. In ROA, this dual configuration enabled practical applications by avoiding intensity variations associated with switching the incident helicity. In the present nonlinear context, an analogous dual‐CPL approach is adopted: the excitation polarization is fixed, and the circularly polarized components of the emitted SH light are analyzed.

Figure 4f presents the polarization‐resolved SH response of D‐CdTe at stages I and II. For LCP_IN_ and RCP_IN_, the scattered SH signal was analyzed using a rotating quarter‐wave plate followed by a polarizer. Rotation angles 45° and 135° correspond to opposite handedness of the scattered circular polarization, as indicated by the oriented circles in the figure.

At stage I, the SH intensity contrast between these angles shows a clear chiroptical response that reverses with the incident helicity. This contrast is markedly amplified at stage II. The enhancement in SH chiroptical ellipticity seen in Figure 3a is thus corroborated here through polarization analysis using the dual‐CPL configuration. Beyond the helicity dependence, the overall polarization patterns differ strikingly between stages I and II. Stokes‐parameter analysis reveals a substantial increase in the degree of circular polarization (DoP), from 0.2 at stage I to 0.5–0.6 at stage II. Such a pronounced evolution in SH polarization and pattern is consistent with a transformation of the underlying crystallographic symmetry, with both the cracked shell and the CdTe core contributing to the observed response.

## 2 Conclusion

We demonstrate the first observation of second‐harmonic hyper‐Mie optical activity, completing the set of nonlinear chiroptical scattering effects predicted decades ago. We also show that this nonlinear interaction can be used to both drive and monitor a material transformation, following in real time the conversion of CdTe/CdO nanohelices into CdO NPs through changes in second‐harmonic intensity, ellipticity, and polarization.

Because the optical signal directly reflects the state of the material, the transformation can be tracked continuously and correlated with specific structural changes. In particular, the onset of shell cracking and exposure of CdTe is marked by a sharp increase in second‐harmonic intensity and a reversal of the chiroptical contrast. This provides a clear experimental signature of the transition and a way to interrupt the process at a chosen stage. The observed shift from hyper‐Mie to hyper‐Rayleigh scattering also links the nonlinear optical response to the evolving length scale of the system.

Natural next steps are to test whether the same closed‐loop chiroptical photochemical scheme can be extended to other materials and to other classes of nonlinear photochemistry, in particular two‐photon polymerization, photo‐release of biologically active molecules, and bond cleavage. It will also be important to determine whether the CdTe Z‐band emission that appears at stage II carries a chiral polarization signature, and whether related closed‐loop behavior can be achieved at the third harmonic, where the fundamental lies further into the infrared. More generally, polarization‐resolved harmonic measurements, ideally correlated with intermediate morphology and with single‐particle transformations, may clarify how structural chirality, crystallographic symmetry, and effective scatterer size evolve during illumination. It will also be very interesting to determine whether circular polarization affects the reaction kinetics. This can be tested by comparing oxidation rates under opposite helicities for a fixed nanohelix handedness, and by examining mixed ensembles to see whether preferential conversion leads to kinetic resolution. These measurements would establish whether the chiroptical interaction demonstrated here can be extended to enantioselective photochemistry.

## 3 Methods

### 3.1 CdTe NP Synthesis and Helix Assembly

Cadmium telluride (CdTe) NPs were synthesized and assembled into mesoscale helices following previously published methods [17].

### 3.2 Synthesis

0.985 g of Cd(ClO_4_)_2_·6 H_2_O and 0.990 g of L‐ or D‐cysteine hydrochloride monohydrate were dissolved in 100 mL of deionized water, and the pH of the mixture was adjusted to 11.2 with 1 m sodium hydroxide (NaOH). Then the solution was placed into a three‐neck round‐bottomed flask and purged with N_2_ for 30 min. H_2_Te gas generated by the reaction of 0.1 g of Al_2_Te_3_ and 10 mL of 0.5 m H_2_SO_4_ was slowly purged into the dispersion. The reaction was then refluxed at 100°C under N_2_ for 10 h to obtain CdTe NPs.

### 3.3 Electron Microscopy

Scanning electron microscopy was acquired on a Thermo Fisher Nova 200 at an accelerating voltage of 5 kV. Scanning transmission electron microscopy (STEM) imaging and EDS elemental analysis were obtained on a Thermo Fisher Talos F200X G2 S/TEM operating at 200 kV.

### 3.4 Assembly

The as‐synthesized CdTe NPs dispersed in water were first aged at room temperature in the dark for 1 day. After initial aging, 40 microliters of 0.1 m Cd^2+^ ion solution (created by dissolving cadmium perchlorate hexahydrate (Cd(ClO_4_)_2_·6 H_2_O) in deionized water) is added per 1 mL of the CdTe NPs dispersed in water. The pH of the solution was then adjusted to 8 with 1:10 HCl solution. The pH adjusted solution was then divided into 2 mL microcentrifuge tubes, with the remaining volume filled with methanol in either a 1:3 or 1:4 CdTe NP solution:methanol ratio. The solutions were then stored under light for three days. Resulting helix samples following assembly were washed three times in deionized water via centrifugation (3 min, 6000 rpm) and redispersed in 0.5 mL of deionized water for CD characterization.

### 3.5 CD Characterization

A J‐1700 CD spectrophotometer equipped with a PMT detector with a 200–800 nm range and an InGaAs NIR detector with an 800–1600 nm range was used for CD studies. Scanning parameters used were as follows: temperature, 25°C; scanning speed, 500 nm/min; data pitch, 1 nm; bandwidth, 1 nm (NIR bandwidth, 20 nm); digital integration time, 0.25 s; and one accumulation.

### 3.6 Sample Preparation

CdTe samples were originally in a solution of 2 mL methanol in an Eppendorf tube. To remove the methanol and replace with water, the CdTe‐Methanol mixture was centrifuged for 3 min at 6000 rpm, separating the CdTe from the methanol. The methanol was then removed via pipette and then 2 mL of distilled water was added to the tube before centrifuging again for 3 min to redisperse the CdTe in the distilled water. Of the resulting mixture, 0.1 mL was added to a beaker with 1.9 mL of distilled water to dilute the mixture. 1 mL of this final mixture was placed in a cuvette for measurements.

### 3.7 Experimental Measurements

Nonlinear measurements at 730 and 710 nm were performed with a Chameleon VisionS Ti:Sapphire laser with an 80 MHz rep rate and 75 fs pulse duration. The 800 nm measurements were performed with a Mai Tai SP Ti:Sapphire laser. A chopper with a 3.3% duty cycle and a frequency of 42 Hz was used along with a two‐channel gated photon counter and with a Hamamatsu photomultiplier tube to detect photons in the UV wavelengths. Circularly polarized light was produced by an achromatic quarter waveplate with design wavelength 400–800 nm. Laser power was controlled using a GL10 Glan laser polarizer oriented to allow S‐polarized light and a half waveplate. A long pass filter with a cut‐off wavelength of 665 nm was used to block out any residual UV light that may have been generated in prior optics. Light was focused into a fused quartz cuvette by an achromatic lens with a focal length of 30 mm. The scattered light was collimated by an antireflection coated lens with a focal length of 25.4 mm and focused into the PMT by a lens with focal length 200 mm. A band pass filter in the range 335–610 nm was placed after the collimating lenses to block the fundamental beam. An additional band pass filter with optical density 4+ was placed in front of the PMT.

For the polarization measurements, an analyzing quarter waveplate (AWP) and a second polarizer oriented to allow the horizontal (P) direction were placed after the collimation lens. The AWP was rotated between 0 and 315° in 45° steps. The input power was approximately 6.1 mW and an incident wavelength of 730 nm was used.

Long‐duration measurements (up to ∼1.5 × 10^5^ s) were repeated multiple times, yielding consistent temporal evolution (see Supporting Information). Experiments were performed in a temperature‐ and humidity‐controlled laboratory with minimal air flow. Samples were contained in sealed, single‐piece quartz cuvettes (without glued pieces) to minimize evaporation and to avoid long‐term optical or chemical degradation, including potential contamination from glue. Control measurements showed no systematic drift in the optical response over comparable timescales, indicating that the observed dynamics arise from the photo‐induced transformation. The laser stability vs. time is illustrated in Figure S10.

## Conflicts of Interest

The authors declare no conflicts of interest.

## Supporting information




**Supporting File**: adma73593‐sup‐0001‐SuppMat.docx.

## Data Availability

The data that support the findings of this study are openly available in the repository of the University of Bath at https://doi.org/10.15125/BAT‐H01613.
