# Supplementary material for: Second‐Harmonic Hyper‐Mie Optical Activity Enables Closed‐Loop Chiral Photochemistry
Source: Adv Mater. 2026 Jun 11;38(40):e73593. doi: 10.1002/adma.73593 (PMC13378259; doi:10.1002/adma.73593)
Supplement: Supplementary file 1 — Supporting File: adma73593‐sup‐0001‐SuppMat.docx. [file ADMA-38-e73593-s001.docx]

**Title: Second-Harmonic Hyper-Mie Optical Activity Enables Closed-Loop Chiral Photochemistry**

**Authors:** Hoyeon Choi^1^, Kody Wisnant,^2,3^ Ben J. Olohan^1^, E. Petronijevic,^4^ G. Dan Pantoș,^5^ Nicholas A. Kotov,^2,3^ Ventsislav K. Valev^1,6^

**Affiliations:**

^1^ Centre for Photonics, Department of Physics, University of Bath, Bath, BA2 7AY, United Kingdom.

^2^ Department of Chemical Engineering, University of Michigan, Ann Arbor, MI, USA

^3^ Biointerfaces Institute, University of Michigan, Ann Arbor, MI, USA.

^4^ SBAI Department, La Sapienza University of Rome, Rome, Italy

^5^ Department of Chemistry, University of Bath, Bath, BA2 7AY, United Kingdom.

^6^ Department of Electronic & Electrical Engineering, University of Bath, Bath BA2 7AY, United Kingdom.

*Corresponding authors. Email: [v.k.valev@bath.ac.uk](mailto:v.k.valev@bath.ac.uk), [kotov@umich.edu](mailto:kotov@umich.edu)

**Fig. S1** presents the setup for optical measurements at the second-harmonic and in the linear optical regime.


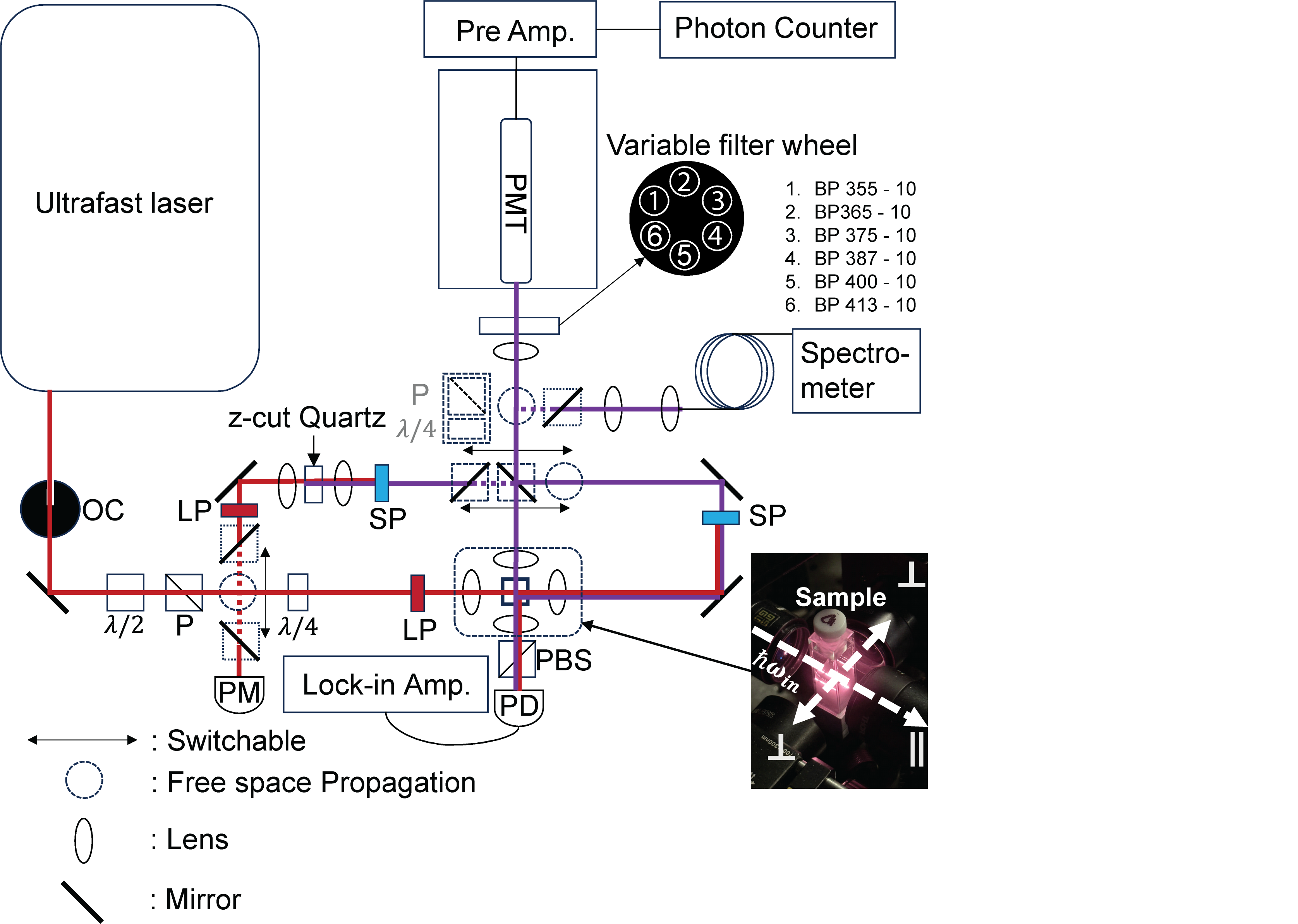


**Fig. S1: Experimental setup diagram for the optical measurements.**

OC: optical chopper; PMT: photomultiplier tube; MS: mechanical shutter; PD: photodiode; PBS: polarizing beam splitter; P: polarizer; λ/2: half-wave plate; λ /4: quarter-wave plate; LP: long pass filter; SP: short pass filter; BP: band pass filter; PM: Power meter.


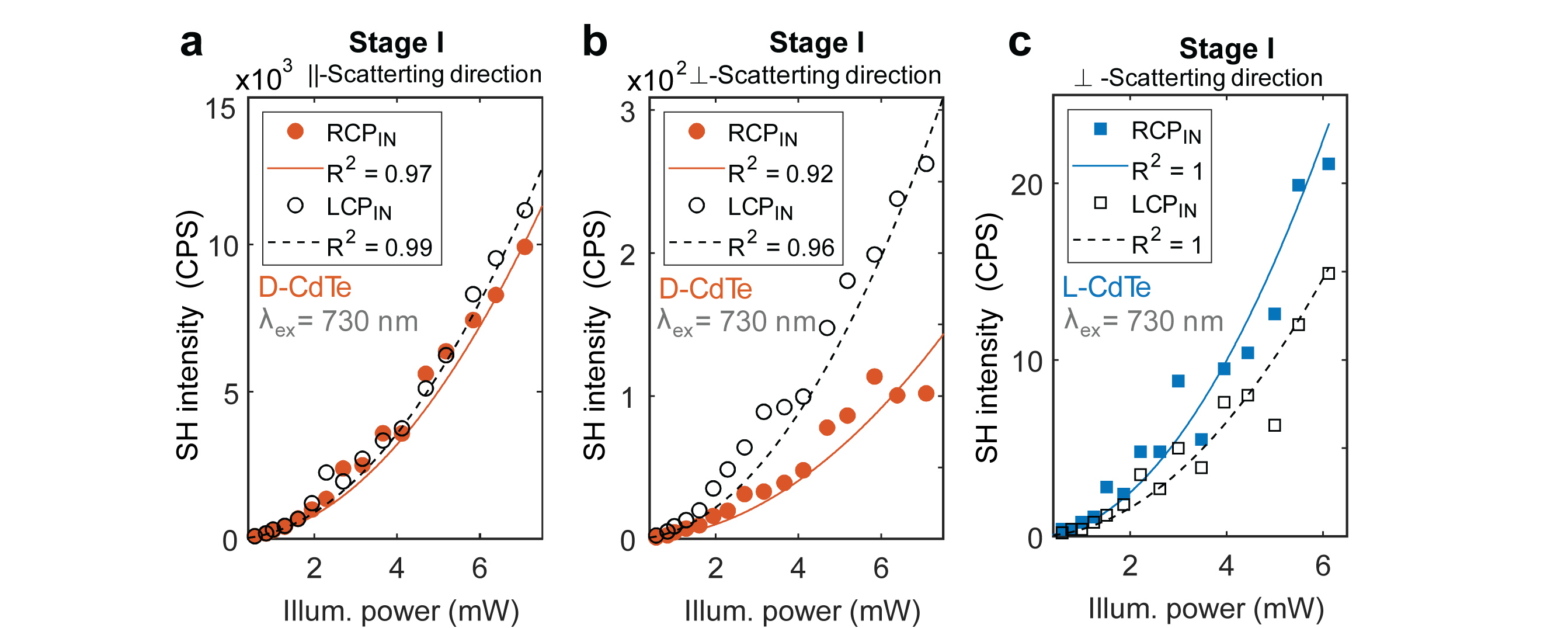


**Fig. S2: Power dependence of second-harmonic (SH) scattered light for incident right-hand circularly polarized (RCP_IN_) and left-hand circularly polarized (LCP_IN_) light, at 730 nm. a**, Forward scattering SH intensity from D-CdTe. **b**, Right-angled SH scattering intensity from D-CdTe. **c**, Right-angled SH scattering intensity from L-CdTe. The symbols are experimental data, and the lines are quadratic fits, with R^2^ provided.


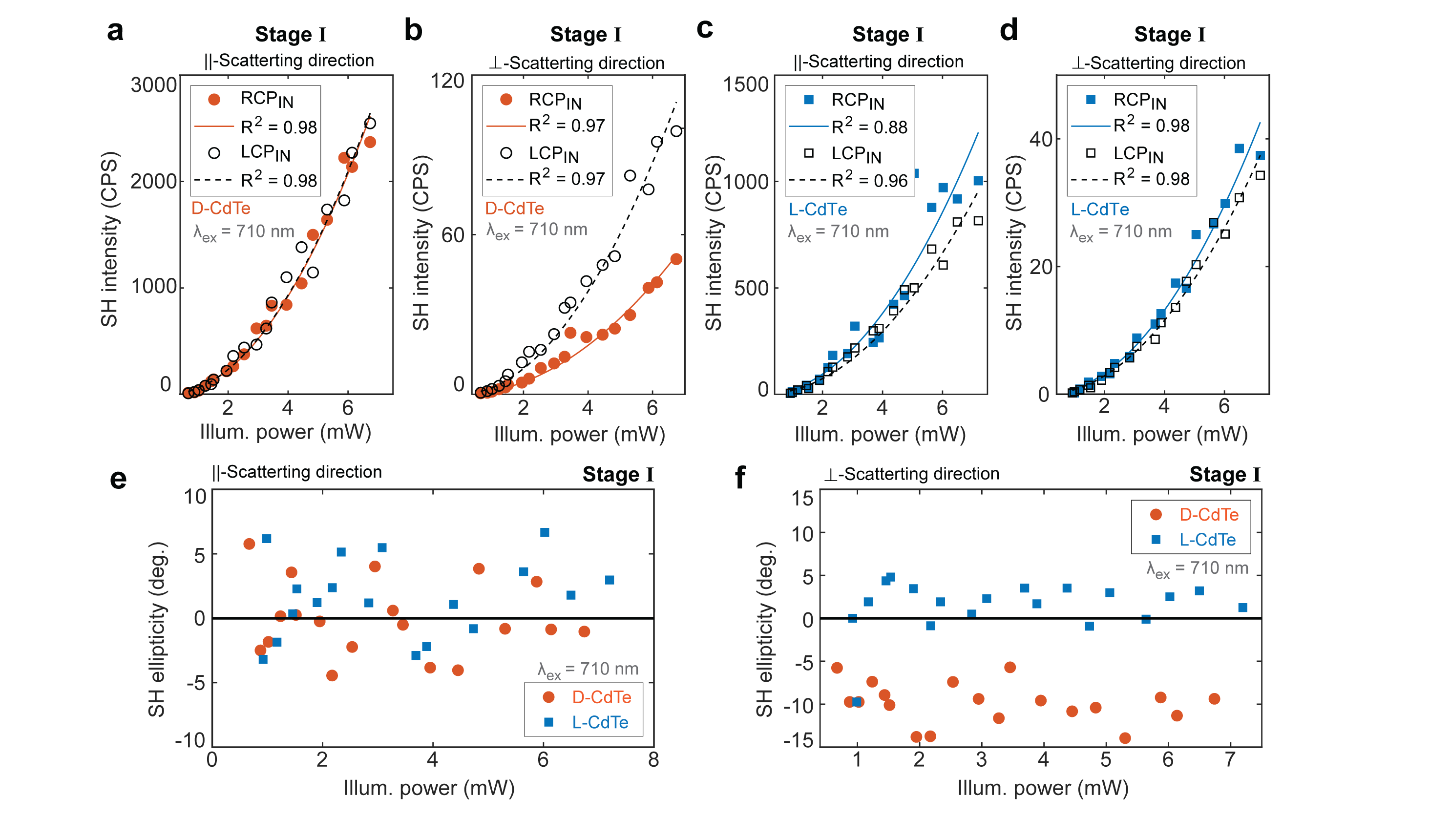


**Fig. S3: Power dependence of SH scattered light for illumination at 710 nm with RCP_IN_ and LCP_IN_.**

**a**, Forward scattering SH intensity from D-CdTe. **b**, Right-angled scattering SH intensity from D-CdTe. **c**, Forward scattering SH intensity from L-CdTe. **d**, Right-angled scattering SH intensity from L-CdTe. The symbols are experimental data, and the lines are quadratic fits, with R^2^ provided. **e**, correspondent SH ellipticity of D/L-CdTe in the forward direction and, **f**, in the right-angled direction.


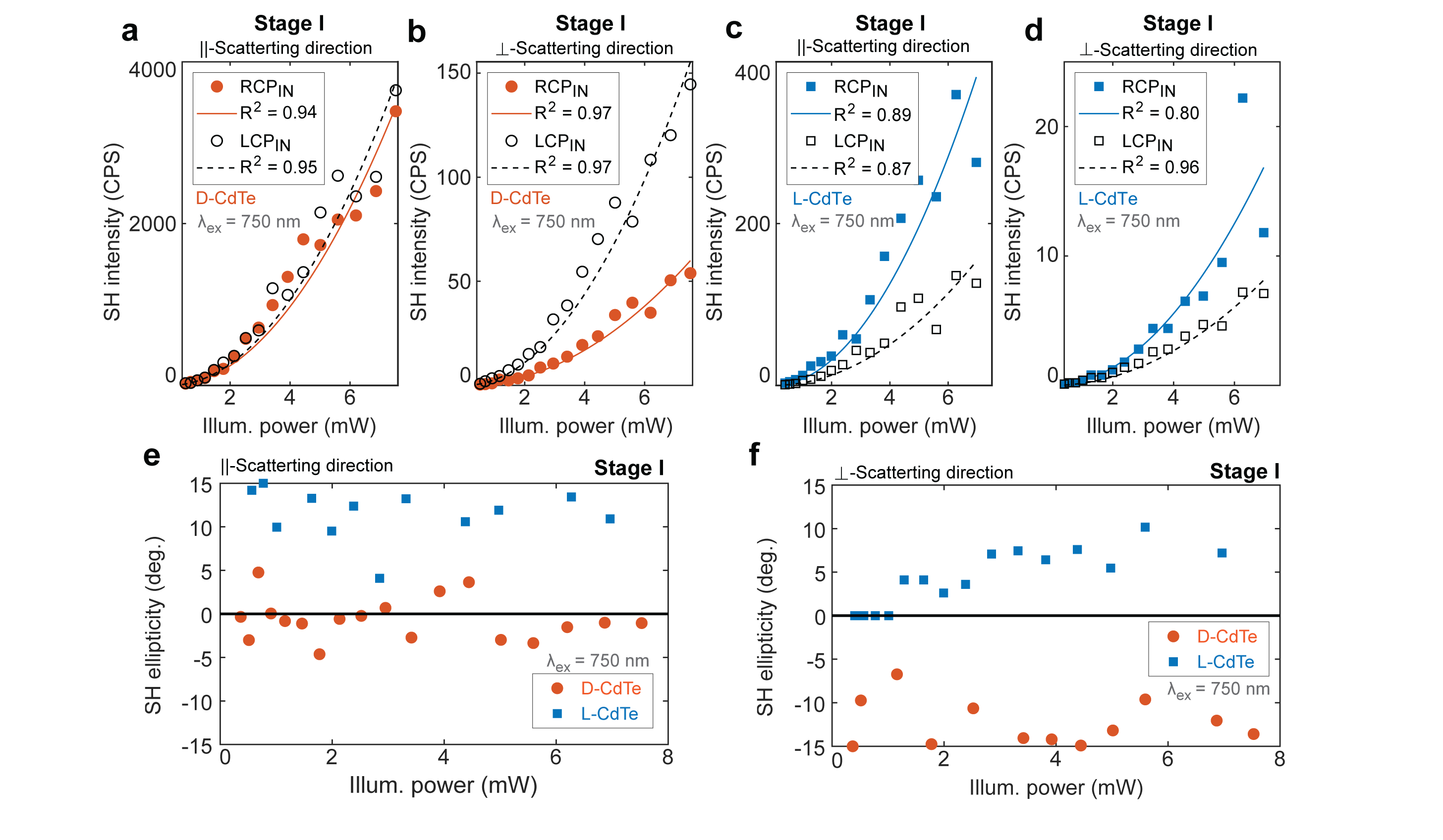


**Fig. S4: Power dependence of SH scattered light for illumination at 750 nm with RCP_IN_ and LCP_IN_.**

**a**, Forward scattering SH intensity from D-CdTe. **b**, Right-angled scattering SH intensity from D-CdTe. **c**, Forward scattering SH intensity from L-CdTe. **d**, Right-angled scattering SH intensity from L-CdTe. The symbols are experimental data, and the lines are quadratic fits, with R^2^ provided. **e**, correspondent SH ellipticity of D/L-CdTe in the forward direction and, **f**, in the right-angled direction.


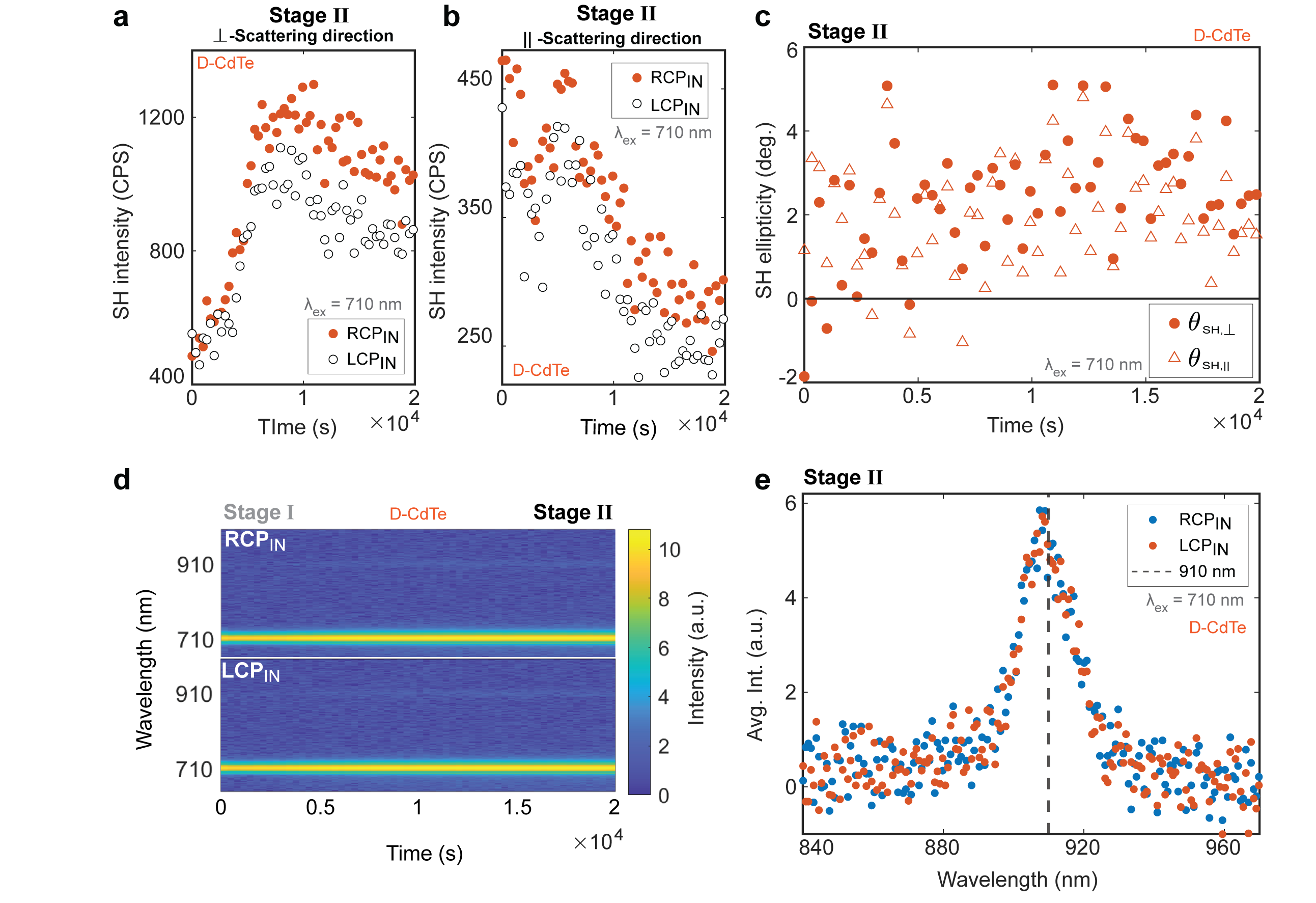


**Fig. S5: Onset of stage II in D-CdTe nanohelices.** Illumination wavelength 710 nm and power 5 mW. **a**, Right-angled SH scattering intensity for RCP_IN_ and LCP_IN_ vs. time. **b**, forward scattering SH intensity scattering vs. time. **c**, The calculated SH ellipticities in the forward and right-angled directions. **d**, Z-band photoluminescence emission for RCP_IN_ and LCP_IN_. **e**, zoom in on the Z-band photoluminescence spectra region – FWHM is centred at ~910 nm.


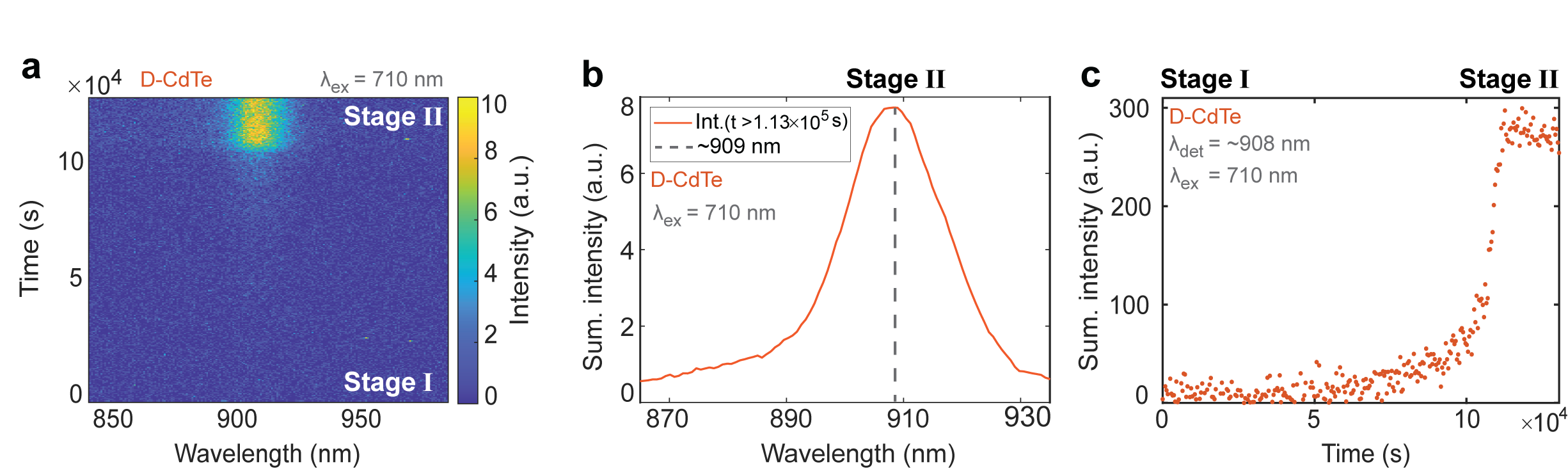


**Fig. S6: Onset of stage II in D-CdTe nanohelices.** Illumination wavelength 710 nm and power 5 mW. **a**, Emission spectrum vs. time, from D-CdTe. **b**, The average spectrum after the onset of the Z-band photoluminescence at ~909 nm. **c**, The sum of detected light intensity in the spectral range between 863 and 937 nm plotted vs. time shows the clear onset of Z-band photoluminescence.


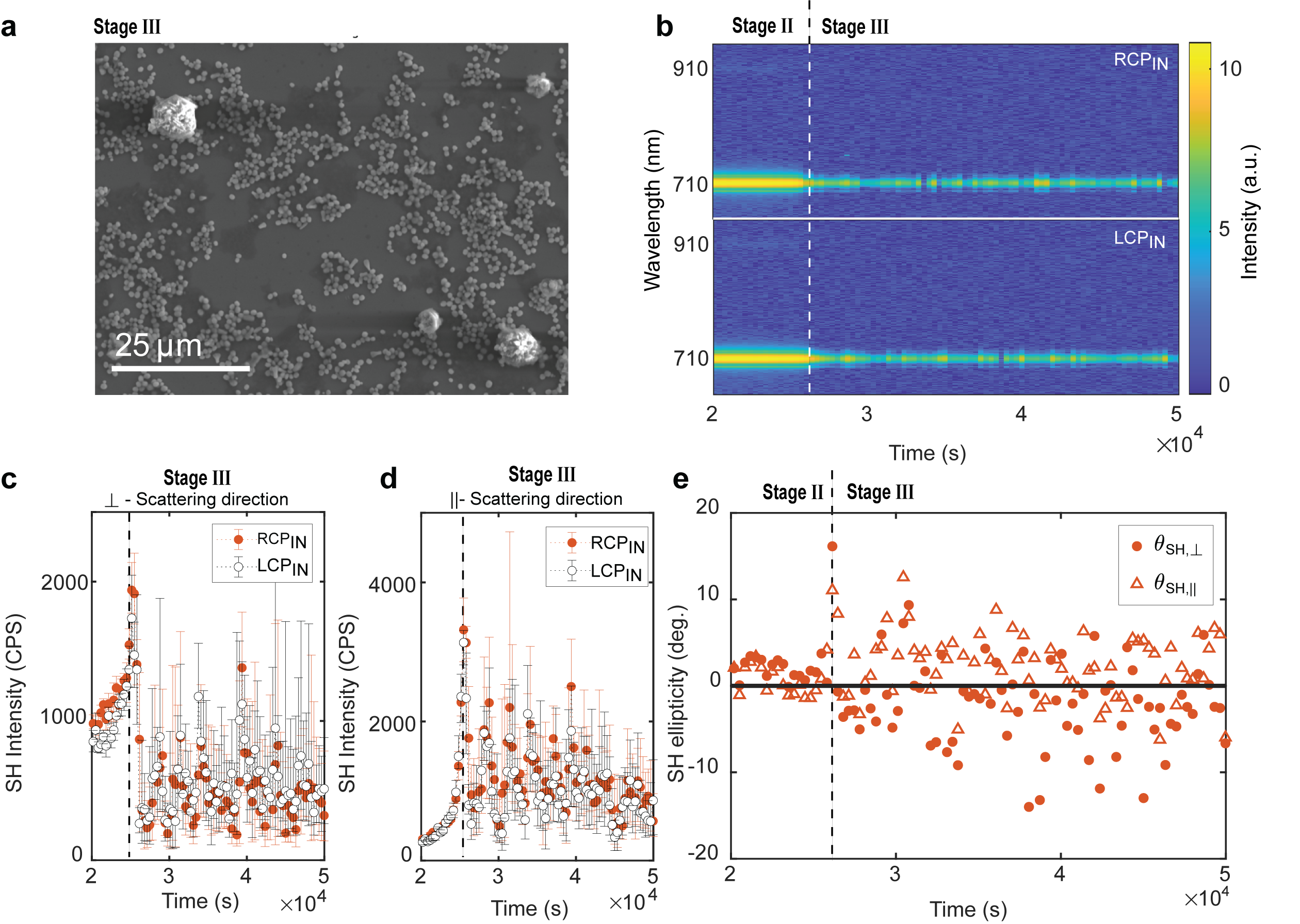


**Fig. S7: Stage II to stage III transition for D-CdTe nanohelices.** **a**, SEM image of the nanoparticles at stage III. **b**, Emission spectrum vs. time, for RCP_IN_ and LCP_IN_. Z-band photoluminescence stops and the scattering intensity at 710 nm drops. **c**, SH intensity vs. time in the right-angled direction. **d**, SH intensity vs. time in the forward direction. **e**, The corresponding SH ellipticity.


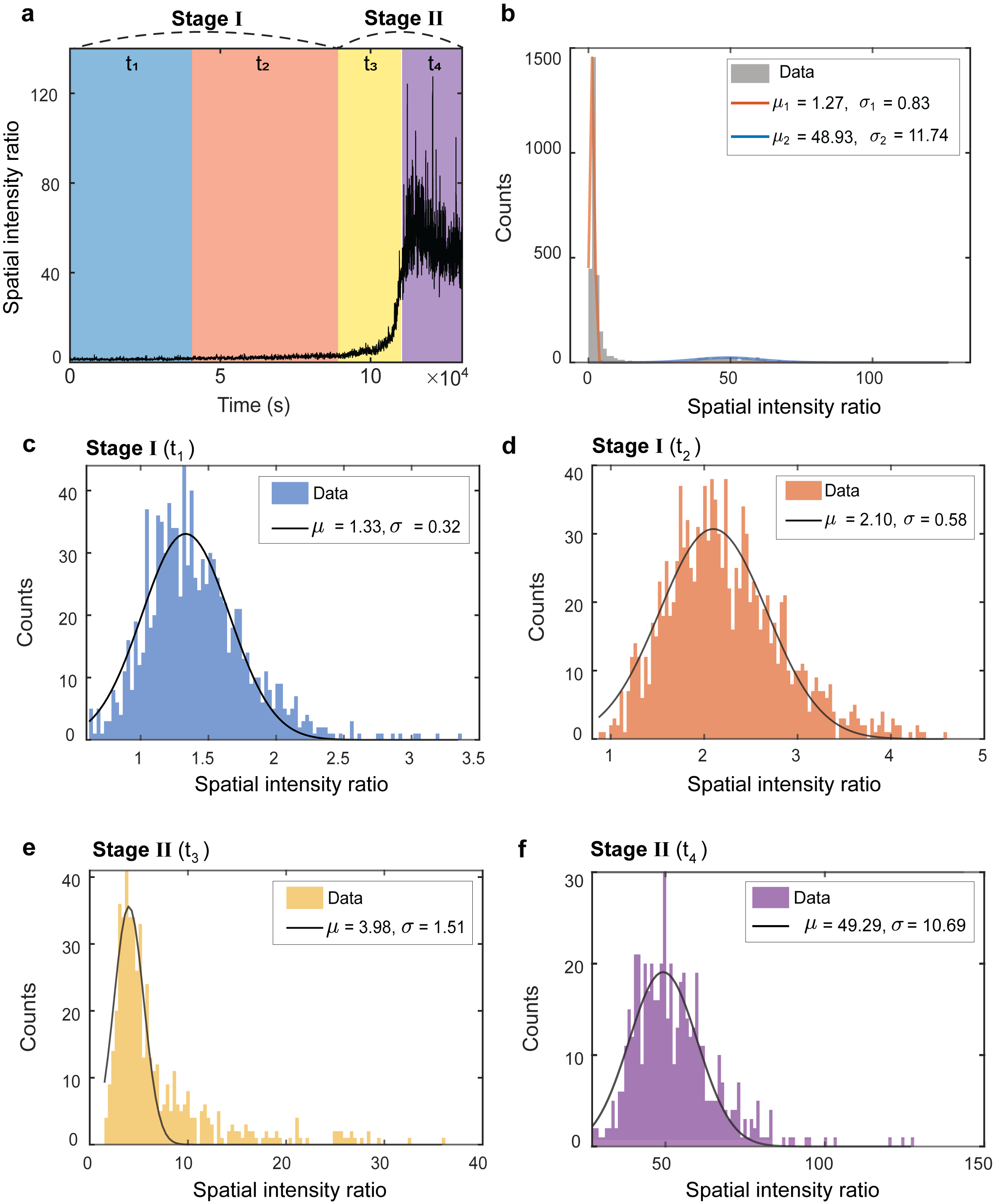


**Fig. S8: Stage I to stage II transition in D-CdTe.** We define the spatial intensity ratio (SIR) as the SH intensity recorded in the right-angled direction divided by the SH intensity recorded in the forward direction. For clarity, we introduce four different time periods, noted t_1_ to t_4_. **a**, the SIR vs time shows the transition from stage I to stage II. **b**, The histogram of all the calculated SIRs is bimodal. **c** to **f**, The histograms of calculated SIR within the periods t_1_ to t_4_, respectively.


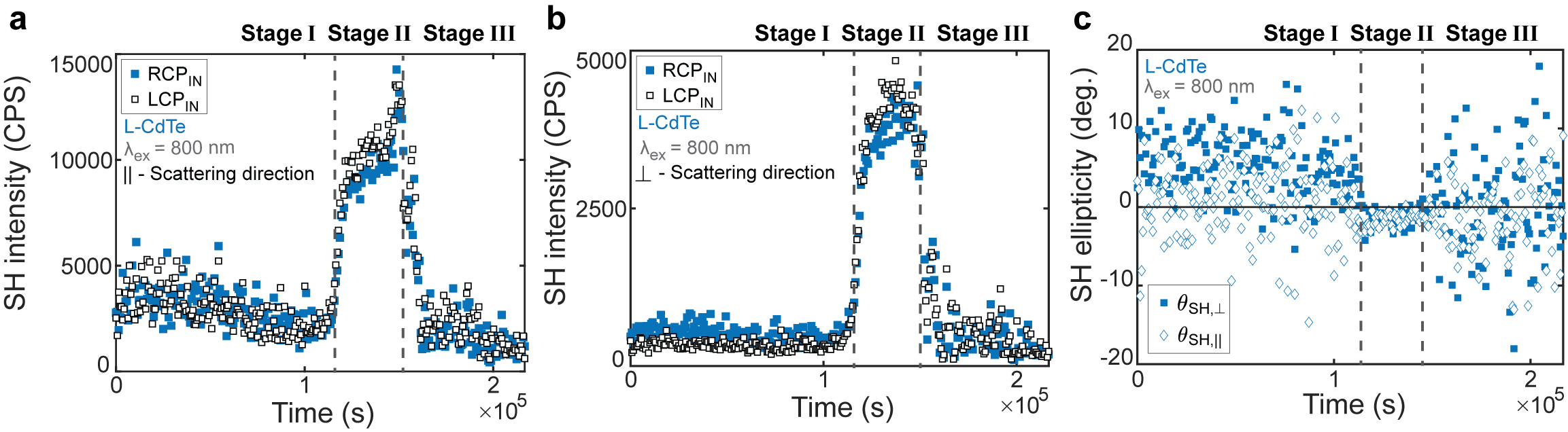


**Fig. S9: Stage I to stage III transitions in L-CdTe.** **a**, Second-harmonic (SH) intensity scattered from L-CdTe nanohelices, as a function of time, for incident right- and left-hand circularly polarized light (RCP_IN_ & LCP_IN_, respectively). Illumination at 800 nm, with ~ 5 mW. Light is detected in the forward (||) direction **b**, similarly organized data for and light detected in the right-angled ($\perp$) direction. **c**, The SH ellipticity vs. time demonstrates the three different stages.


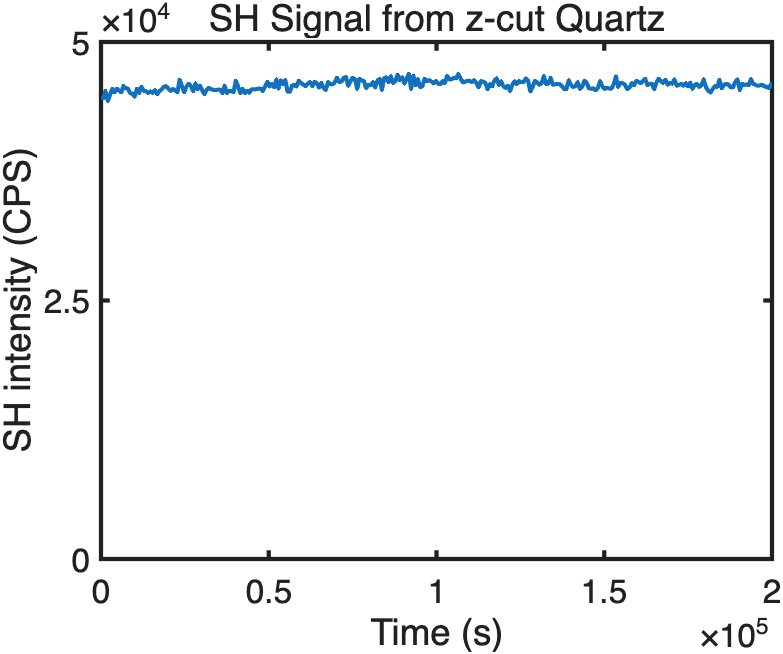


**Fig. S10:** **Second-harmonic intensity (in counts per second) from a reference z-cut single quartz crystal as a function of time.** The data indicates the stability of the laser system during the duration of our experiments both in terms of average power and in terms of pulse shape (both are important for the second-harmonic process.
